# Supplementary material for: Observationally constrained projection of Afro-Asian monsoon precipitation
Source: Nat Commun. 2022 May 10;13:2552. doi: 10.1038/s41467-022-30106-z (PMC9090792; doi:10.1038/s41467-022-30106-z)
Supplement: Supplementary file 1 — Supplementary Information [file 41467_2022_30106_MOESM1_ESM.docx]

**Supplementary Information**

**Observationally constrained projection of Afro-Asian monsoon precipitation**

Chen et al.

**Supplementary Table S1.** Information of CMIP6 30 models.

| **Model** | **Institute/Country** | **Lat x Lon** | **SSP2-4.5** | **SSP5-8.5** | **piControl** | **Runoff** |
| --- | --- | --- | --- | --- | --- | --- |
| ACCESS-CM2 | CSIRO/Australian | 144 x 192 |  | ✓ | ✓ | ✓ |
| ACCESS-ESM1-5 | CSIRO/Australian | 145 x 192 |  | ✓ | ✓ | ✓ |
| AWI-CM-1-1-MR | AWI/Germany | 192 x 384 |  | ✓ | ✓ |  |
| BCC-CSM2-MR | BCC-CMA/China | 160 x 320 | ✓ | ✓ | ✓ | ✓ |
| CAMS-CSM1-0 | CAMS-CMA/China | 160 x 320 | ✓ | ✓ | ✓ | ✓ |
| CESM2-WACCM | NCAR/USA | 192 x 288 |  | ✓ | ✓ | ✓ |
| CNRM-CM6-1 | CNRM-CERFACS/France | 128 x 256 | ✓ | ✓ | ✓ | ✓ |
| CNRM-ESM2-1 | CNRM-CERFACS/France | 128 x 256 | ✓ | ✓ | ✓ | ✓ |
| CanESM5 | CCCMA/Canada | 64 x 128 | ✓ | ✓ | ✓ | ✓ |
| CanESM5-CanOE | CCCMA/Canada | 64 x 128 |  | ✓ | ✓ | ✓ |
| EC-Earth3 | EC-Earth-Consortium/EU | 256 x 512 | ✓ | ✓ | ✓ | ✓ |
| EC-Earth3-Veg | EC-Earth-Consortium/EU | 256 x 512 | ✓ | ✓ | ✓ | ✓ |
| FGOALS-f3-L | LASG-IAP/China | 180 x 360 | ✓ | ✓ | ✓ | ✓ |
| FGOALS-g3 | LASG-IAP/China | 90 x 180 | ✓ | ✓ | ✓ | ✓ |
| GFDL-CM4 | GFDL-NOAA/USA | 180 x 360 | ✓ | ✓ | ✓ | ✓ |
| GFDL-ESM4 | GFDL-NOAA/USA | 180 x 360 | ✓ | ✓ |  | ✓ |
| GISS-E2-1-G | GISS-GISS/USA | 90 x 144 |  | ✓ | ✓ | ✓ |
| HadGEM3-GC31-LL | MOHC/UK | 144 x 192 |  | ✓ | ✓ | ✓ |
| INM-CM4-8 | INM/Russia | 120 x 180 |  | ✓ | ✓ | ✓ |
| INM-CM5-0 | INM/Russia | 120 x 180 | ✓ | ✓ | ✓ | ✓ |
| IPSL-CM6A-LR | IPSL/France | 143 x 144 | ✓ | ✓ | ✓ | ✓ |
| MCM-UA-1-0 | UA/USA | 80 x 96 |  | ✓ | ✓ | ✓ |
| MIROC6 | MIROC/Japan | 128 x 256 | ✓ | ✓ | ✓ | ✓ |
| MIROC-ES2L | MIROC/Japan | 64 x 128 | ✓ | ✓ | ✓ | ✓ |
| MPI-ESM1-2-HR | MPI-M/Germany | 192 x 384 |  | ✓ | ✓ | ✓ |
| MPI-ESM1-2-LR | MPI-M/Germany | 96 x 192 |  | ✓ | ✓ | ✓ |
| MRI-ESM2-0 | MRI/Japan | 160 x 320 |  | ✓ | ✓ | ✓ |
| NESM3 | NUIST/China | 96 x 192 | ✓ | ✓ | ✓ |  |
| NorESM2-LM | NCC/Norway | 96 x 144 |  | ✓ | ✓ | ✓ |
| UKESM1-0-LL | MOHC/UK | 144 x 192 | ✓ | ✓ | ✓ | ✓ |

**Supplementary Table S2.** Information of observational datasets

| **Surface temperature** | **Resolution** | **Time range** | **Reference** |
| --- | --- | --- | --- |
| Berkeley Earth Surface Temperature (BEST) | 1º x 1º | 1701-present | Rohde^1^ |
| Cowtan and Way version 2 (v2) | 5º x 5º | 1850-present | Cowtan and Way^2^ |
| NASA Goddard Institute for Space Studies Surface Temperature version 5 (GISTEMP) | 2º x 2º | 1800-present | Lenssen^3^ |
| NOAA Global Surface Temperature version 5 (NOAAGlobalTemp v5) | 5º x 5º | 1880-present | Vose^4^ and Zhang^5^ |
| **Precipitation** | **Resolution** | **Time range** | **Reference** |
| Climatic Research Unit (CRU) Time-Series version 4.02 | 0.5º x 0.5º | 1901-present | Harris^6^ |
| Global Precipitation Climatology Centre version 7 (GPCC v7) | 1º x 1º | 1901-present | Schneider^7^ |
| Climate Prediction Center (CPC) Merged Analysis of Precipitation (CMAP v1201) | 2.5º x 2.5º | 1979-present | Xie and Arkin^8^ |
| Global Precipitation Climatology Project (GPCP v2.2) | 2.5º x 2.5º | 1979-present | Adler^9^ |

**Supplementary Table S3.** The interhemispheric thermal contrast index ($ITC_{I}$) in the historical simulation of 30 CMIP6 models and in the observation for the period of 1981-2014. The red boxes denote that the simulated $ITC_{I}$ exceed the range of the observation. The observed $ITC_{I}$ is shown by the mean of multiple observation datasets and the uncertainty range contributed from multiple observation datasets and internal variability.

| **Models and Observation** | $\boldsymbol{IT}\boldsymbol{C}_{\boldsymbol{I}}$ **((34K yr^-1^)^2^)** |
| --- | --- |
| Observation | 0.12$\pm$0.03 |
| ACCESS-CM2 | 0.18 |
| ACCESS-ESM1-5 | 0.23 |
| AWI-CM-1-1-MR | 0.19 |
| BCC-CSM2-MR | 0.16 |
| CAMS-CSM1-0 | 0.10 |
| CESM2-WACCM | 0.25 |
| CNRM-CM6-1 | 0.13 |
| CNRM-ESM2-1 | 0.13 |
| CanESM5 | 0.30 |
| CanESM5-CanOE | 0.33 |
| EC-Earth3 | 0.29 |
| EC-Earth3-Veg | 0.18 |
| FGOALS-f3-L | 0.14 |
| FGOALS-g3 | 0.19 |
| GFDL-CM4 | 0.22 |
| GFDL-ESM4 | 0.14 |
| GISS-E2-1-G | 0.10 |
| HadGEM3-GC31-LL | 0.21 |
| INM-CM4-8 | 0.12 |
| INM-CM5-0 | 0.14 |
| IPSL-CM6A-LR | 0.20 |
| MCM-UA-1-0 | 0.13 |
| MIROC6 | 0.14 |
| MIROC-ES2L | 0.12 |
| MPI-ESM1-2-HR | 0.10 |
| MPI-ESM1-2-LR | 0.12 |
| MRI-ESM2-0 | 0.14 |
| NESM3 | 0.24 |
| NorESM2-LM | 0.21 |
| UKESM1-0-LL | 0.25 |


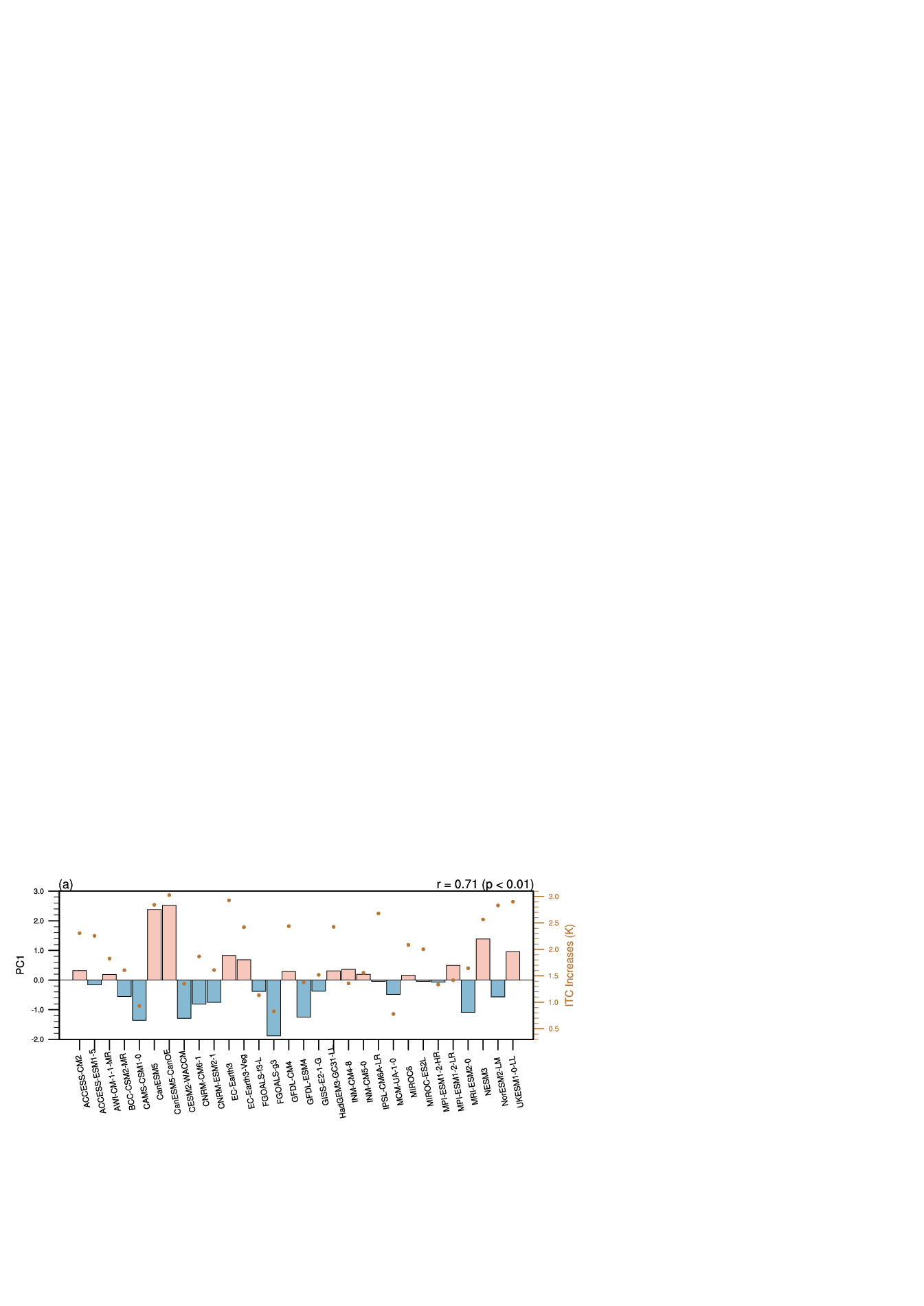


**Supplementary Figure S1.** **Normalized principal components (PC1, bar) and projected interhemispheric thermal contrast (ITC, dot, K; see Methods) changes.** The normalized inter-model PC1 and the projected interhemispheric thermal contrast changes in 2050~2099 relative to 1965~2014 are shown. The correlation coefficient between PC1 and ITC with significant level is shown on the top right corner.


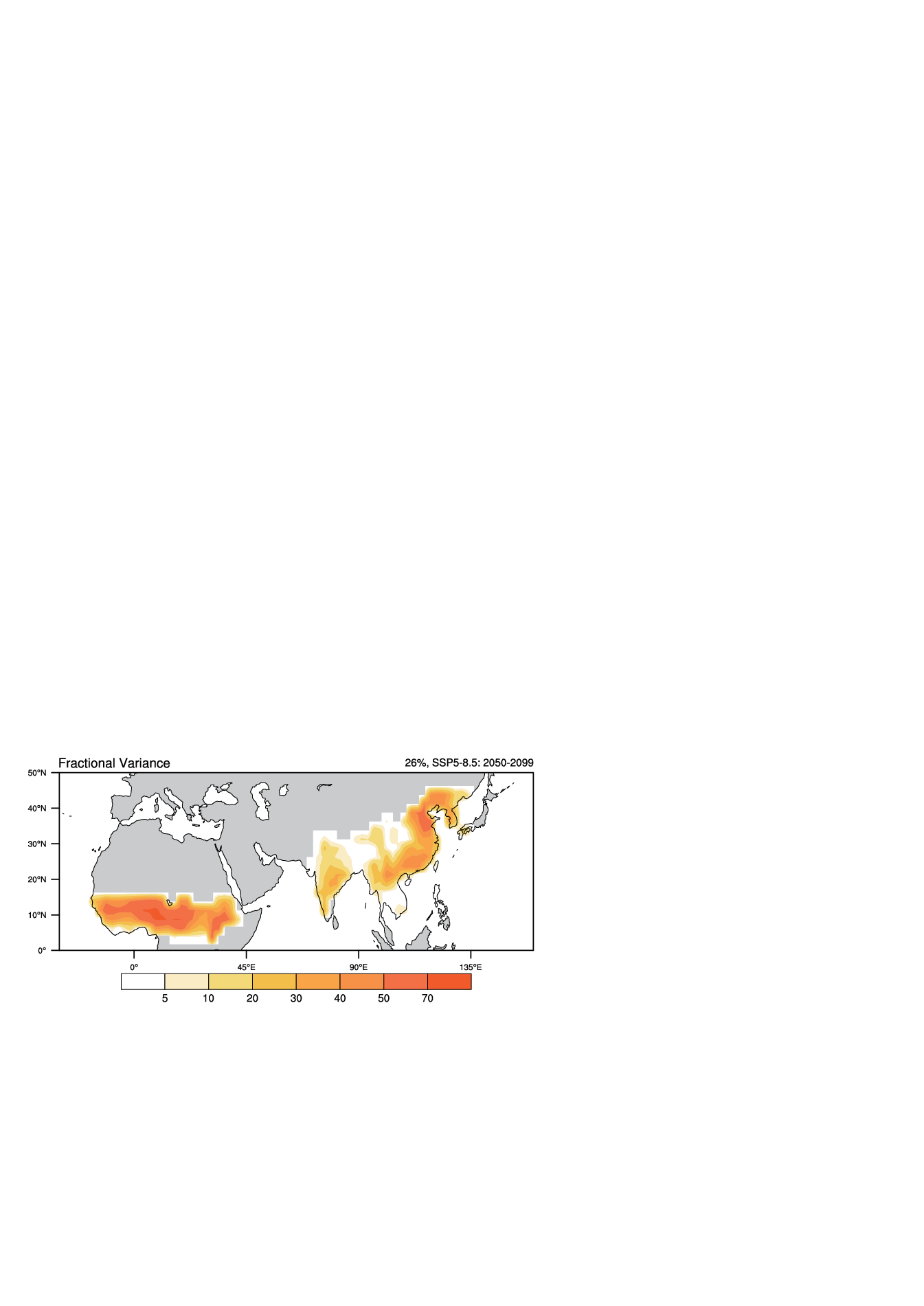


**Supplementary Figure S2.** **Fractional variances accounted by the leading mode of the projection of the Afro-Asian summer monsoon (AfroASM) precipitation**. The leading mode are derived from the inter-model empirical orthogonal function (EOF) analysis of projected changes of the AfroASM precipitation (see Methods). The percentage on the top-right corner is explained inter-model variance.


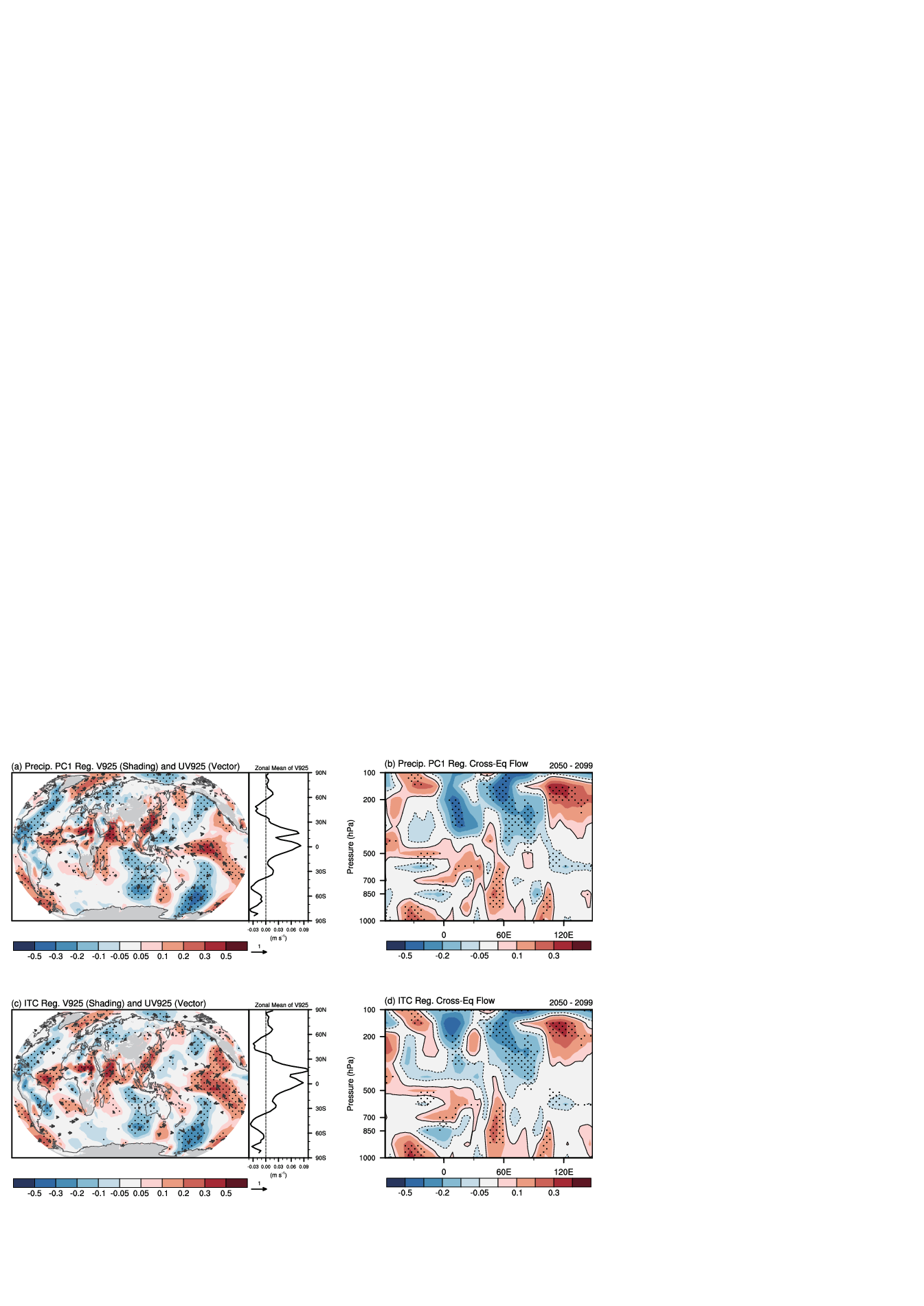


**Supplementary Figure S3**. **The low-level cross equator flow related to PC1 and projected interhemispheric thermal contrast (ITC).** (a) The anomalies of the meridional wind (shading) and the horizontal circulation (vector) in the low-level troposphere (925 hPa, units: m s^-1^) and (b) the longitude-height cross section of the meridional wind (4ºS~4ºN, units: m s^-1^) in 2050-2099 relative to 1965-2014 across models regress onto the normalized inter-model PC1. (c) and (d) is same as (a) and (b) but for the regression onto the normalized ITC changes across models in 2050-2099. The stippling and vectors denote the regression exceeds 90% confidence level. The right panels in (a) and (c) are the zonal mean of the low-level meridional wind related to PC1 and ITC, respectively.


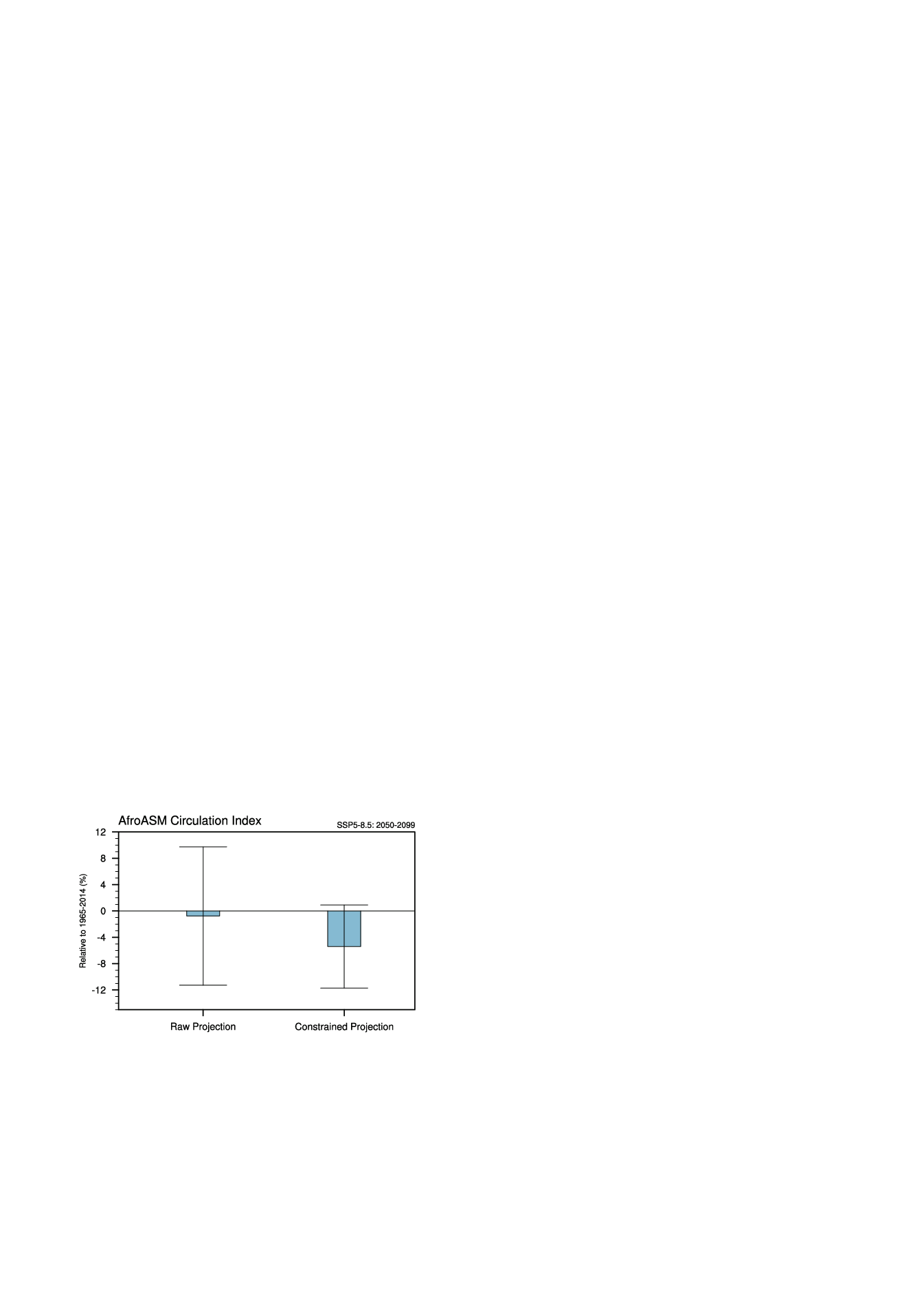


**Supplementary Figure S4.** **Projected changes in the Afro-Asian summer monsoon (AfroASM) circulation index of unconstrained (“Raw Projection”) and constrained projection**. The bars represent the multi-model ensemble, while the vertical lines indicate the range of $\pm$1$\sigma$ across models. For more details of the definition of circulation index, please see Methods.


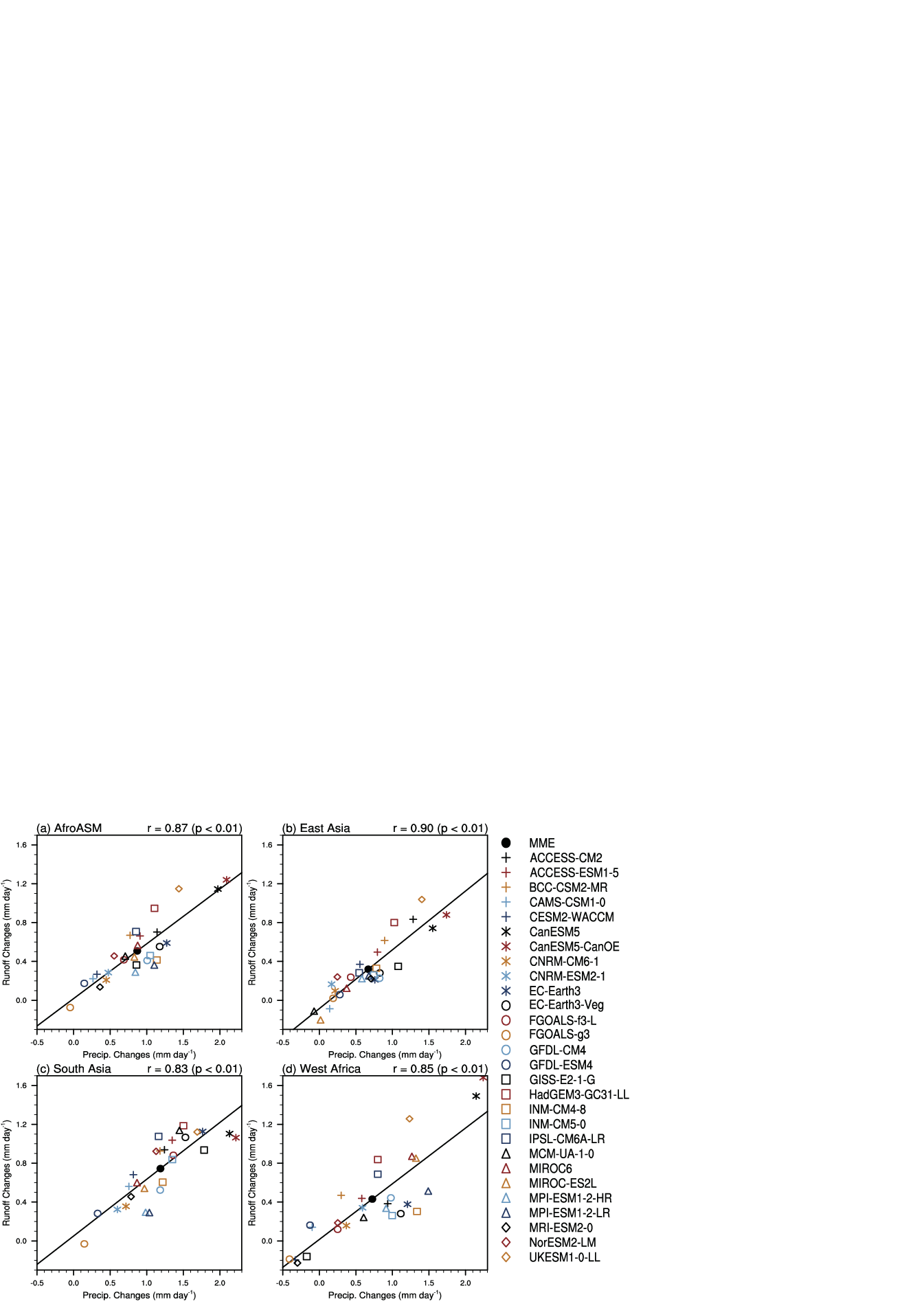


**Supplementary Figure S5**. **Relationship between projected changes of precipitation and runoff** in 2050~2099 under SSP5-8.5 scenario averaged over averaged over (a) Afro-Asian (AfroASM), (b) East Asia, (c) South Asia and (d) West Africa monsoon regions. The solid lines indicate the linear fit, with correlation coefficient and significant level based on student t-test shown on the top-right corner.


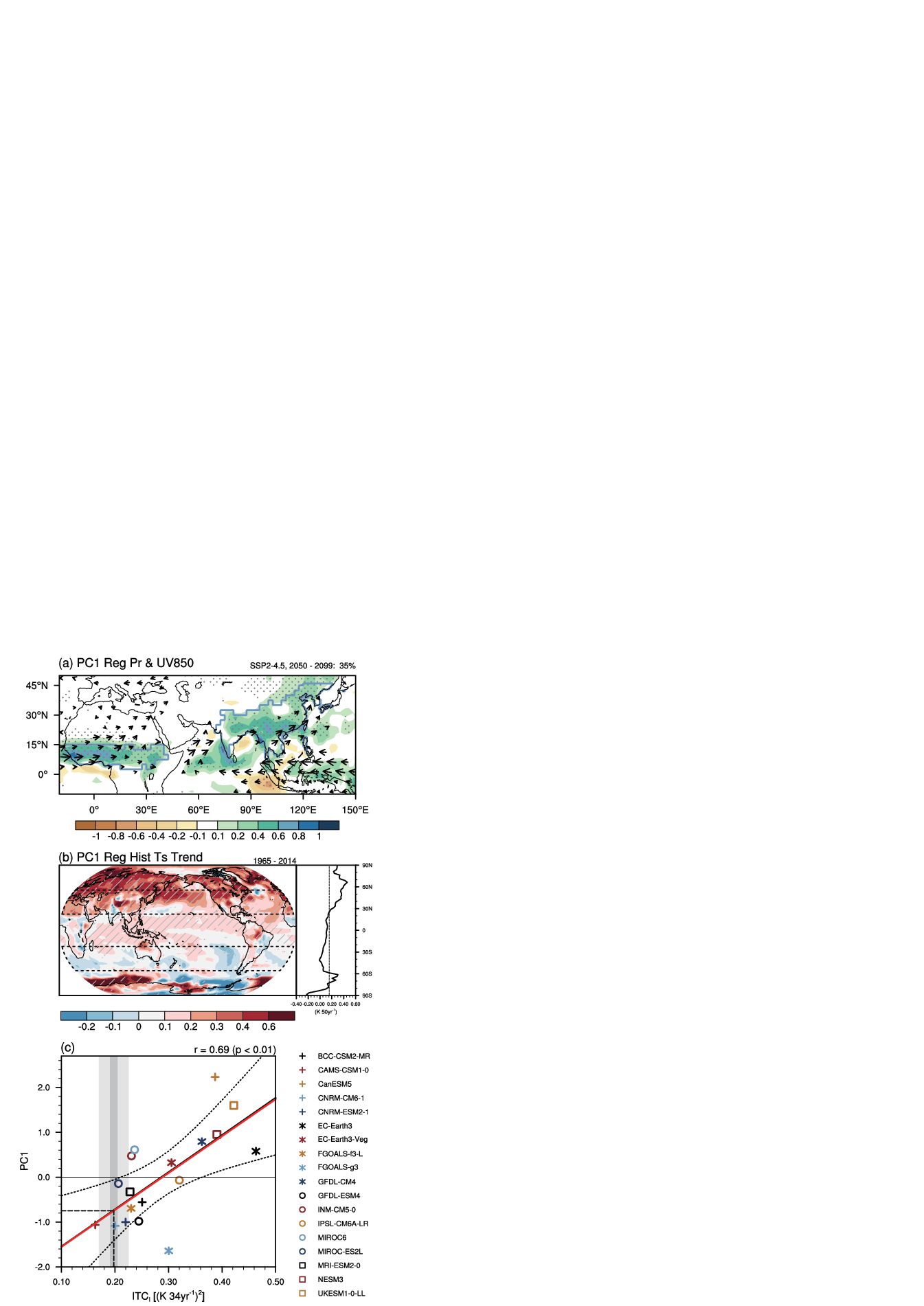


**Supplementary Figure S6.** Same as Figure 2a, Figure 2c, Figure 2e and Figure 4b, but for the results under the SSP2-4.5 scenario.


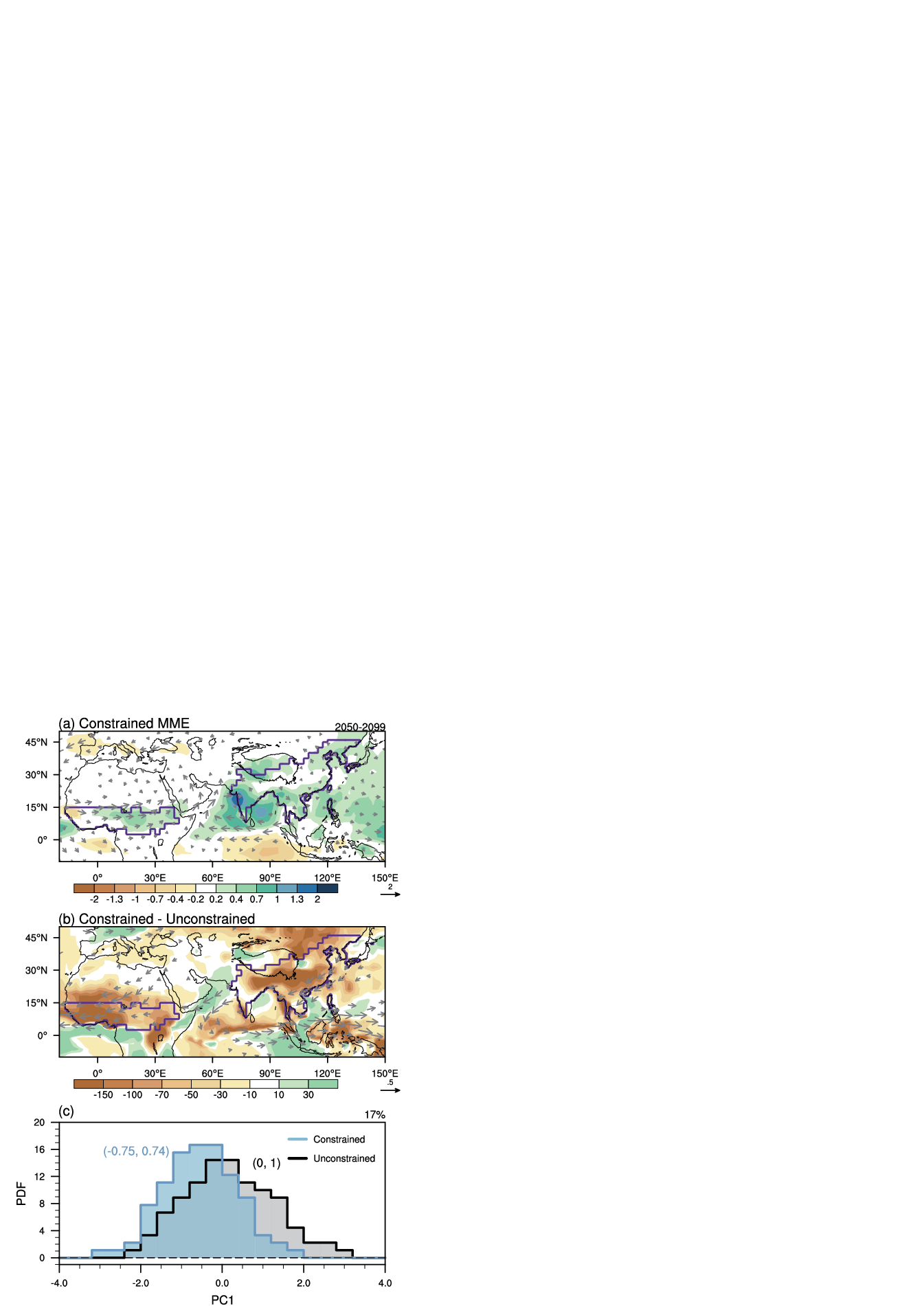


**Supplementary Figure S7.** Same as Figure 5 but for the results under the SSP2-4.5 scenario.

**Supplementary Information Note #1:**

We use different model ensembles to verify the robustness of the emergent constraint. Firstly, we deduct one to four models from the 30 CMIP6 models, and we get 30 ($C_{30}^{1}=30$), 435 ($C_{30}^{2}=435$), 4060 ($C_{30}^{3}=4060$) and 27405 ($C_{30}^{4}=27405$) subsets of model ensemble in total, respectively. Then, we calculate the relationship between $ITC_{I}$ and PC1 in each subset. The results are shown as the blue bars in Supplementary Figure S8.

To exam whether the addition of outliers would alter the relationship of emergent constraint, we randomly add one to four outliers to the raw 30-model ensemble (“+1” to “+4” of the red bars in the Supplementary Figure S8). The outliers are created randomly in the range of PC1 and $ITC_{I}$, respectively. We repeat the above processes 1000 times to form 1000 synthetic members. The results are shown as the red bars in Supplementary Figure S8.

The robustness is defined as the range of correlation coefficient in different subsets of model ensemble exceeding the thresholds of the 5% significant level under student t-test.


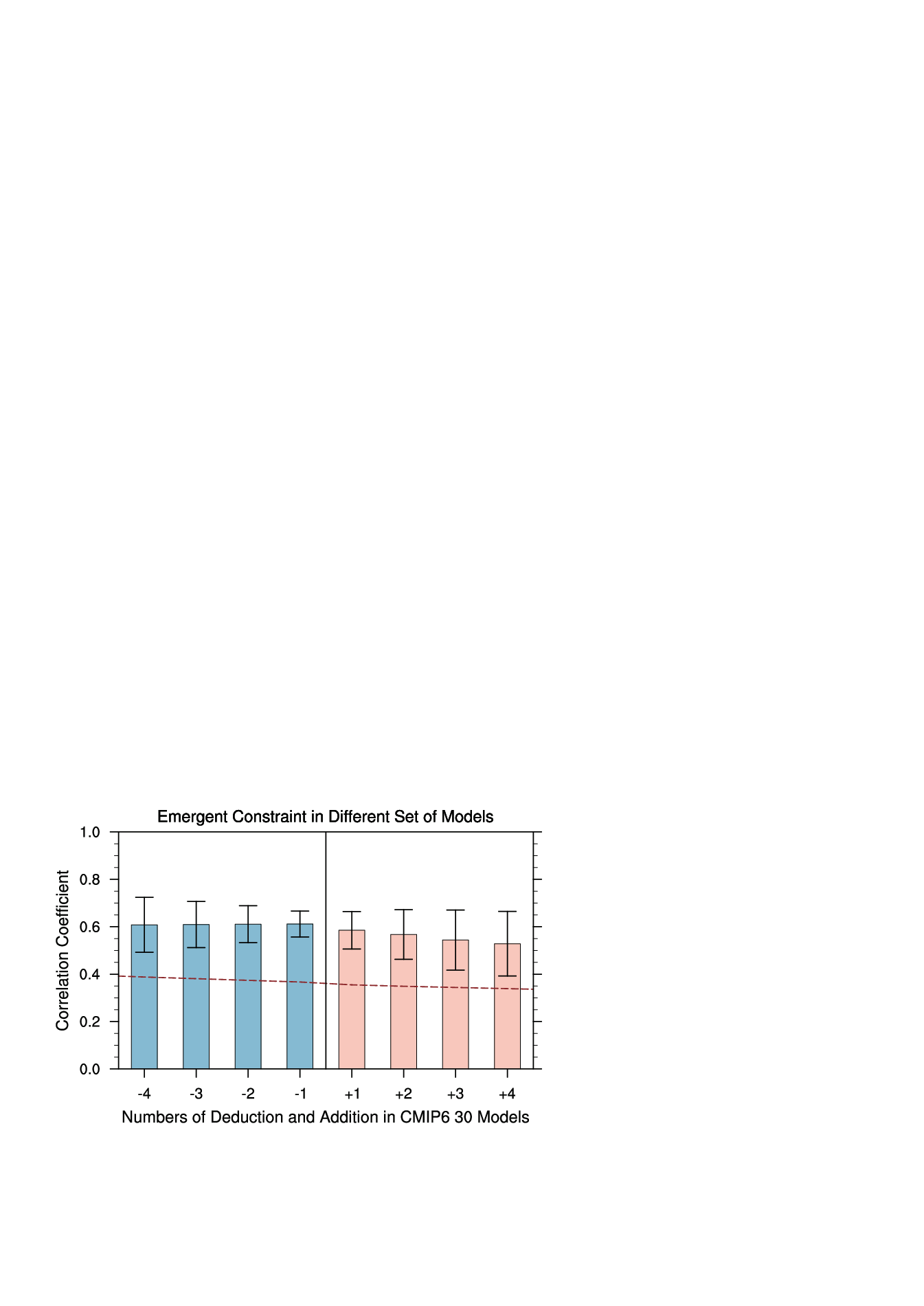


**Supplementary Figure S8. Robustness test for the emergent constraint using different sets of model ensembles.** The relationship is represented by the inter-model correlation coefficient between interhemispheric thermal contrast pattern index ($ITC_{I}$) and normalized PC1. The bar charts and the error bars represent the ensemble mean and range of 5th -95th percentile of correlation coefficient across different set of models. The methods on how to create different sets of models is described in Supplementary Information Note #1. The red dash curve shows the thresholds of the 5% significant level under the student t-test.


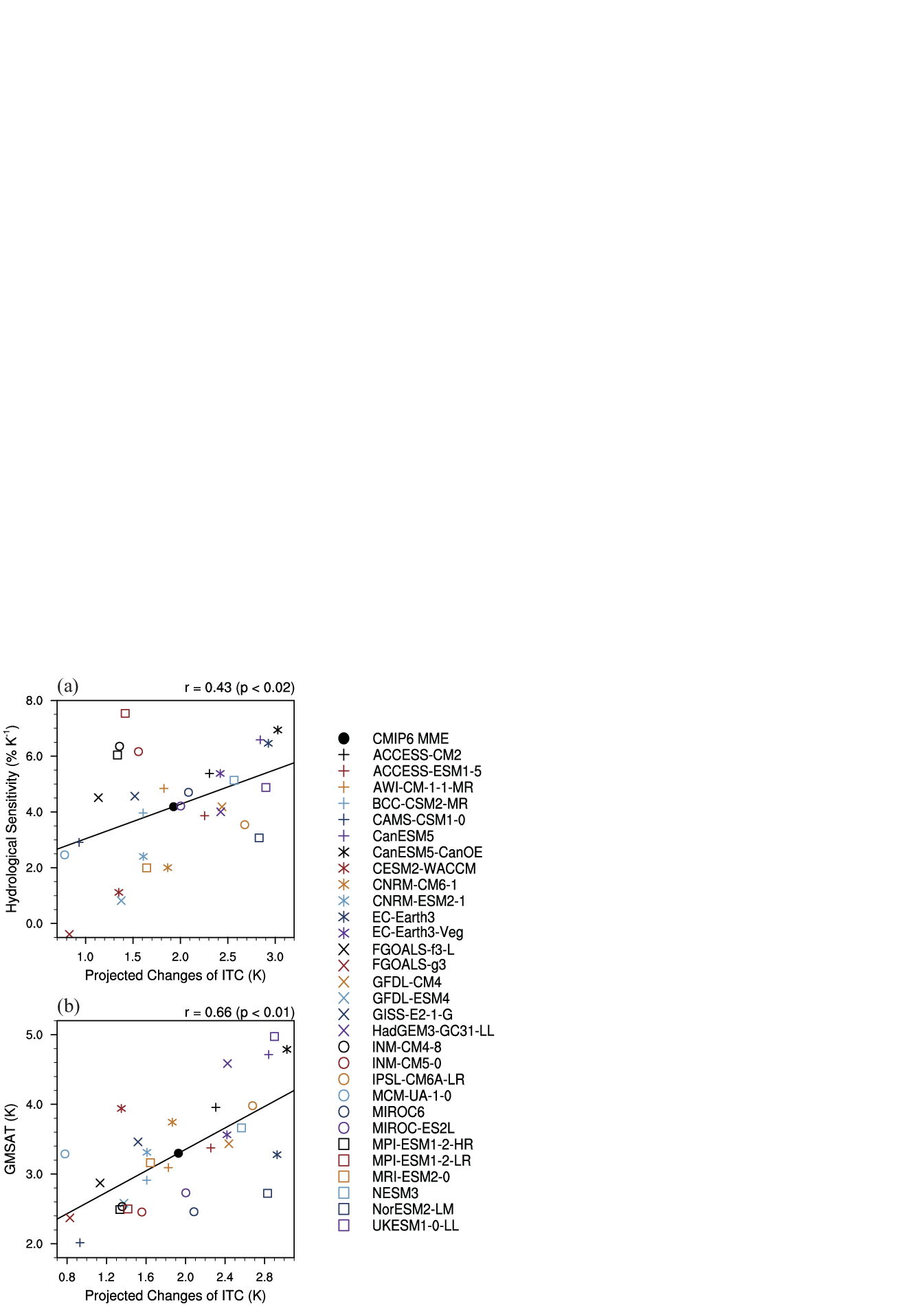


**Supplementary Figure S9. Inter-model relationship between the projection changes of interhemispheric thermal contrast (ITC, K) and the hydrological sensitivity (% K^-1^), and between the projection changes of ITC and global mean surface air temperature (GSAT, K) warming.** The change and the warming are represented by the anomaly in 2050-2099 relative to 1965-2014. The results on the top-right corner are the correlation coefficient and significant level under Student t-test.

**Supplementary References:**

1. Rohde, R., Muller, R., Jacobsen, R., Perlmutter, S. & Mosher, S. Berkeley Earth temperature averaging process. *Geoinformatics Geostatistics An Overv.* **01**, (2013).

2. Cowtan, K. & Way, R. G. Coverage bias in the HadCRUT4 temperature series and its impact on recent temperature trends. *Q. J. R. Meteorol. Soc.* **140**, 1935–1944 (2014).

3. Lenssen, N. *et al.* Improvements in the GISTEMP uncertainty model. *J. Geophys. Res. Atmos.* **124**, 6307–6326 (2019).

4. Vose, R. S. *et al.* NOAA’s merged land-ocean surface temperature analysis. *Bull. Am. Meteorol. Soc.* **93**, 1677–1685 (2012).

5. Zhang, H.-M. *et al.* Updated temperature data give a sharper view of climate trends. *Eos (Washington. DC).* **100**, (2019).

6. Harris, I., Jones, P. D., Osborn, T. J. & Lister, D. H. Updated high-resolution grids of monthly climatic observations - the CRU TS3.10 Dataset. *Int. J. Climatol.* **34**, 623–642 (2014).

7. Schneider, U. *et al.* GPCC’s new land surface precipitation climatology based on quality-controlled in situ data and its role in quantifying the global water cycle. *Theor. Appl. Climatol.* **115**, 15–40 (2013).

8. Xie, P. & Arkin, P. A. Global precipitation: a 17-year monthly analysis based on gauge observations, satellite estimates, and numerical model outputs. *Bull. Am. Meteorol. Soc.* **78**, 2539–2558 (1997).

9. Adler, R. F. *et al.* The version-2 global precipitation climatology project (GPCP) monthly precipitation analysis (1979-present). *J. Hydrometeorol.* **4**, 1147–1167 (2003).
